# Surgical approach and the impact of epidural analgesia on survival after esophagectomy for cancer: A population-based retrospective cohort study

**DOI:** 10.1371/journal.pone.0211125

**Published:** 2019-01-22

**Authors:** Kenneth C. Cummings III, Tzuyung Doug Kou, Amitabh Chak, Mark D. Schluchter, Seunghee Margevicius, Gregory S. Cooper, Neal J. Meropol, Yaron Perry, Philip A. Linden, Linda C. Cummings

**Affiliations:** 1 Anesthesiology Institute, Cleveland Clinic, Cleveland, Ohio, United States of America; 2 Case Comprehensive Cancer Center, Cleveland, Ohio, United States of America; 3 Department of Anesthesiology, Case Western Reserve University School of Medicine, Cleveland, Ohio, United States of America; 4 Department of Quantitative and Population Health Sciences, Case Western Reserve University, Cleveland, Ohio, United States of America; 5 Department of Medicine, University Hospitals Cleveland Medical Center, Cleveland, Ohio, United States of America; 6 Department of Medicine, Case Western Reserve University School of Medicine, Cleveland, Ohio, United States of America; 7 Department of Surgery, University Hospitals Cleveland Medical Center, Cleveland, Ohio, United States of America; 8 Department of Surgery, Case Western Reserve University School of Medicine, Cleveland, Ohio, United States of America; University Hospital Hamburg Eppendorf, GERMANY

## Abstract

**Background:**

Esophagectomy for esophageal cancer carries high morbidity and mortality, particularly in older patients. Transthoracic esophagectomy allows formal lymphadenectomy, but leads to greater perioperative morbidity and pain than transhiatal esophagectomy. Epidural analgesia may attenuate the stress response and be less immunosuppressive than opioids, potentially affecting long-term outcomes. These potential benefits may be more pronounced for transthoracic esophagectomy due to its greater physiologic impact. We evaluated the impact of epidural analgesia on survival and recurrence after transthoracic versus transhiatal esophagectomy.

**Methods:**

A retrospective cohort study was performed using the linked Surveillance, Epidemiology and End Results (SEER)-Medicare database. Patients aged ≥66 years with locoregional esophageal cancer diagnosed 1994–2009 who underwent esophagectomy were identified, with follow-up through December 31, 2013. Epidural receipt and surgical approach were identified from Medicare claims. Survival analyses adjusting for hospital esophagectomy volume, surgical approach, and epidural use were performed. A subgroup analysis restricted to esophageal adenocarcinoma patients was performed.

**Results:**

Among 1,921 patients, 38% underwent transhiatal esophagectomy (n = 730) and 62% underwent transthoracic esophagectomy (n = 1,191). 61% (n = 1,169) received epidurals and 39% (n = 752) did not. Epidural analgesia was associated with transthoracic approach and higher volume hospitals. Patients with epidural analgesia had better 90-day survival. Five-year survival was higher with transhiatal esophagectomy (37.2%) than transthoracic esophagectomy (31.0%, p = 0.006). Among transthoracic esophagectomy patients, epidural analgesia was associated with improved 5-year survival (33.5% epidural versus 26.5% non-epidural, p = 0.012; hazard ratio 0.81, 95% confidence interval [0.70, 0.93]). Among the subgroup of esophageal adenocarcinoma patients undergoing transthoracic esophagectomy, epidural analgesia remained associated with improved 5-year survival (hazard ratio 0.81, 95% confidence interval [0.67, 0.96]); this survival benefit persisted in sensitivity analyses adjusting for propensity to receive an epidural.

**Conclusion:**

Among patients undergoing transthoracic esophagectomy, including a subgroup restricted to esophageal adenocarcinoma, epidural analgesia was associated with improved survival even after adjusting for other factors.

## Introduction

Approximately 17,290 new esophageal cancer cases are expected in the U.S. in 2018, with 15,850 deaths.[1] The histologic subtype of most new cases in the U.S. is adenocarcinoma; its incidence has been rising over the past several decades.[2] With a median age of 68 at diagnosis,[3] esophageal cancer has a 5-year disease-free survival of <40% after treatment with resection alone,[4, 5] reflecting early spread and recurrence. While the addition of neoadjuvant therapy has led to improvements in 5-year progression-free survival to 44%, prognosis for this disease remains poor.[6] Survival after recurrence is 6–12 months,[5, 7, 8] occurring a median of 10–12 months postoperatively.[4, 7, 8]

Esophagectomy is potentially curative but carries high morbidity and mortality,[9, 10] particularly with a transthoracic approach (TTE). The transhiatal approach (THE) may reduce perioperative mortality,[11] but precludes a full thoracic lymphadenectomy. Mortality after esophagectomy is associated with lower hospital esophagectomy volume.[12, 13] Esophagectomy results in circulating tumor cells which may promote subsequent metastasis.[14] Perioperative factors including surgery,[15] the stress response,[16] blood transfusion,[17] opioids,[18] and general anesthesia[19] impair immune function. TTE may suppress immune function more substantially than THE through a greater reduction in T-helper type 2 cytokine production,[15] while epidural analgesia (EA) may be less immunosuppressive than opioids by achieving superior pain control, thereby attenuating the surgical stress response.[20] EA may also support immune function by reducing systemic opioid exposure. Surgical approach and EA may therefore modulate immune activity, potentially impacting long-term outcomes through effects on tumor surveillance.

We previously reported that EA is associated with improved survival for colorectal cancer.[21] We hypothesized that EA improves long-term outcomes in esophageal cancer. We therefore aimed to assess the impact of EA on survival and recurrence after esophagectomy, with adjustment for surgical approach due to the potential impact of surgical approach on immune function.

## Materials and methods

A retrospective cohort study was performed using the Surveillance, Epidemiology, and End Results tumor registry linked to billing data from Medicare, a U.S. federal government insurance program that primarily benefits individuals aged ≥65 years (SEER-Medicare). Approval was obtained from the University Hospitals Cleveland Medical Center’s Institutional Review Board and the National Cancer Institute (NCI). Informed consent was not obtained because the data did not contain personal identifiers, and the patients whose data were being reviewed had already been seen, treated, and released from medical care.

### Data sources

SEER provides cancer incidence and survival from registries currently covering approximately 28% of the U.S. population. The geographic areas covered by SEER are reflective of the demographics of the general population in the U.S.[22] SEER data include demographics, tumor site, histology, surgical stage, survival, and initial treatment. Linkage to billing data from Medicare allows identification of comorbidities and treatment after the first 4 months. Surgical resection can be identified from SEER and from Medicare inpatient billing data or Medicare bills from individual physicians. The latter two sources contain International Classification of Diseases, 9^th^ Revision-Clinical Modification (ICD-9-CM) codes and Common Procedural Terminology (CPT) codes, respectively. Common Procedural Terminology codes are common 5-character alphanumeric codes used in insurance claims in the U.S. to designate specific surgical or medical procedures.

### Study population

Patients aged ≥66 years with incident esophageal cancer diagnosed 1994–2009 who underwent esophagectomy were identified. Participation in Medicare inpatient (Part A) and outpatient (Part B) coverage from 6 months before diagnosis until 8 months after diagnosis was required to capture comorbidities and perioperative complications. Patients aged >65 but <66 years were excluded due to lack of data on comorbid conditions; in addition, patients aged <65 years with Medicare were eligible due to disability or end stage renal disease and therefore may have differed in other ways from those in the cohort. Health maintenance organization participants were excluded due to incomplete claims.

### Measures

#### Patient, tumor, and geographic characteristics

Available demographics included age, race, geographic region, gender, and county-level education and income data. Cases with squamous cell carcinoma or adenocarcinoma histology with localized or regional SEER Summary Stage were included. In the SEER staging system, cases are classified as localized (local disease including extension to the muscularis propria or submucosa), regional (direct extension or regional lymph node involvement), or distant (distant lymph nodes or metastasis). Comorbidities were identified using a modified Deyo adaptation of the Charlson comorbidity index[23], excluding malignancy.

#### Esophagectomy and hospital esophagectomy volume

Transthoracic and transhiatal esophagectomies were identified from procedure codes (Appendix). Hospital identifiers in Medicare inpatient or physician-supplier claims were used to calculate hospital esophagectomy volume, ranked into quintiles. Additionally, hospital volume cutpoints specific to each surgical group were determined for sensitivity analyses assessing the impact of EA within each surgical group. Cases missing hospital identifiers were excluded.

#### Analgesia and perioperative transfusion

Thoracic epidural analgesia was identified from CPT codes for epidural placement or management[21, 24, 25] within 7 days of esophagectomy. Patients without these codes were classified in the non-epidural group. Blood transfusion within 7 days of esophagectomy was identified using ICD-9-CM procedure codes.[26]

#### Recurrence

Among patients surviving ≥6 months postoperatively, recurrence was defined by meeting ≥1 of 4 criteria ≥6 months after completion of the initial treatment for esophageal cancer: 1) chemotherapy; 2) radiation; 3) ≥2 Medicare claims containing matching secondary neoplasm ICD-9-CM diagnosis codes (Appendix); or 4) death attributed to esophageal neoplasm.[21, 24, 25] The earliest claim date constituted the recurrence date.

### Statistical analysis

Baseline characteristics by pain management approach and surgical approach were compared using summary statistics. Categorical and continuous variables were compared using chi-square and independent samples t-tests, respectively. P values <0.05 were considered significant. Overall survival (OS) was measured from esophagectomy until death. Time to recurrence was measured starting from 6 months after completion of initial treatment until recurrence; patients dying without known recurrence were censored at time of death. OS and time to recurrence were administratively censored December 31, 2013. Kaplan-Meier curves were generated to assess overall survival (survival cohort) or event-free survival (recurrence cohort) by epidural status. Z-tests were conducted comparing Kaplan-Meier estimates of 5-year survival rates, and log rank tests with censoring at 5 years were used to compare OS and recurrence curves. Median follow-up times were calculated using the Kaplan-Meier estimate of potential follow-up method.[27]

Marginal Cox models were used to adjust for clustering by hospital identifier[28] to assess associations between epidural analgesia and 1) OS and 2) time to recurrence while accounting for surgical approach. Because the test for an interaction for surgical approach with EA was significant in the survival model, marginal Cox models within each surgical group assessing associations between 1) EA and OS and 2) EA and time to recurrence were developed as sensitivity analyses. In addition, survival analyses within each surgical group were performed using propensity score methods. Propensity scores [29] predicting the probability of receiving epidural analgesia were generated from nonparsimonious logistic regression models containing all observed covariates felt to be predictors of use of EA. Marginal Cox models within each surgical group were developed stratifying by propensity score quartile.

To examine the impact of EA on long-term survival without potential for confounding due to histologic subtype, a subgroup analysis restricted to esophageal adenocarcinoma patients was performed. Within this subgroup, in addition to generating marginal Cox models to assess associations between EA and 1) OS and 2) time to recurrence, sensitivity analyses were performed using propensity scores predicting the probability of receiving epidural analgesia.[29] Propensity scores were generated from nonparsimonious logistic regression models containing all observed covariates evaluated in the marginal Cox models. These survival analyses were performed using marginal Cox regression, stratifying on propensity score quartiles.

SAS version 9.3 (Cary, NC) was used for all analyses.

## Results

**Fig 1:**
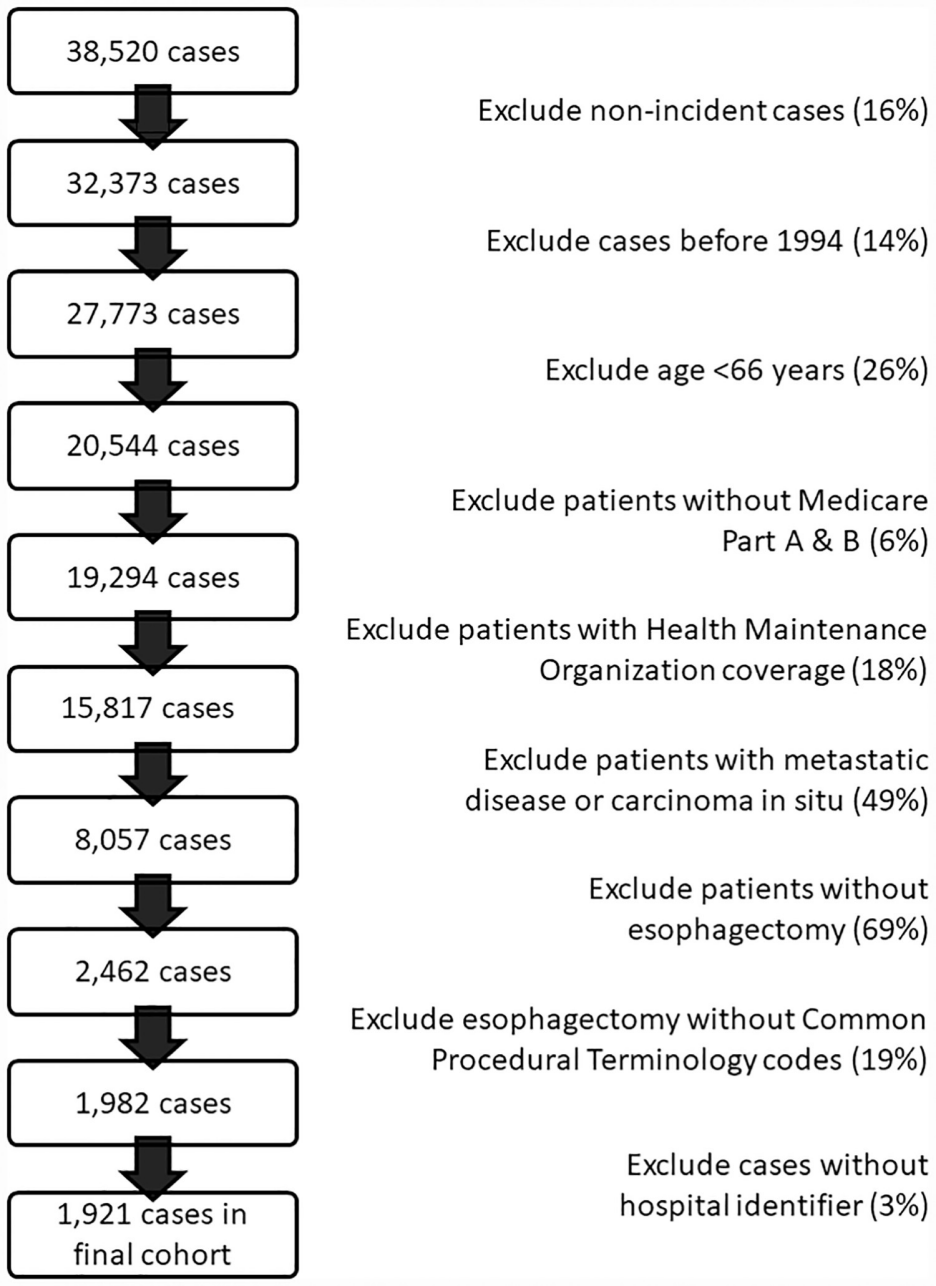
Stepwise identification of cohort. The proportion of patients excluded at each step is provided in parentheses.

We identified 1,921 patients meeting criteria (Fig 1), including 1,191 TTE and 730 THE patients. Baseline characteristics by pain management and surgical approaches are shown (Table 1). EA was given in 60.9% (n = 1,169) of cases, was associated with higher esophagectomy volume (p<0.0001) and was more common with TTE (64.3%, n = 766 vs. 55.2%, n = 403 for THE). THE patients were slightly older and more likely to have localized stage and Charlson score ≥2. Differences in esophagectomy volume were seen with surgical approach (p<0.0001); while TTE was more common with mid-range volume (quintiles 2–4), THE was more common at highest volume hospitals (quintile 5). The median follow-up time in the overall cohort was 2.2 years. Median follow-up time was slightly longer within the THE group (2.5 years) and slightly shorter within the TTE group (2.0 years).

**Table 1:** Baseline characteristics in cohort by pain management approach and surgical approach.

	Pain management approach			Surgical approach		
	No Epidural (n = 752)	Epidural (n = 1,169)	P value	TTE (n = 1,191)	THE (n = 730)	P value
Mean age (SD)	74.0 (5.2)	73.4 (4.9)	0.0483	73.4 (4.9)	74.1 (5.2)	0.0366
Female	168 (22.3)	262 (22.4)	0.9706	260 (21.8)	170 (23.3)	0.4570
Black/Other race	68 (9.0)	91 (7.8)	0.3287	106 (8.9)	53 (7.3)	0.2055
Adenocarcinoma	522 (69.4)	821 (70.2)	0.7035	817 (68.6)	526 (72.1)	0.1088
Localized stage	402 (46.5)	494 (42.3)	0.0648	500 (42.0)	344 (47.1)	0.0275
*Charlson comorbidity score*						
0	234 (31.1)	385 (32.9)	0.1549	404 (33.9)	215 (29.5)	0.0248
1	467 (62.1)	728 (62.3)		731 (61.4)	464 (63.6)	
≥2	51 (6.8)	56 (4.8)		56 (4.7)	51 (7.0)	
Perioperative transfusion	88 (11.7)	117 (10.0)	0.2406	139 (11.7)	66 (9.0)	0.0700
Radiation	301 (40.0)	499 (42.7)	0.2485	505 (42.4)	295 (40.4)	0.3904
*SEER registry region*						
Northeast	174 (23.1)	229 (19.6)	0.0137	270 (22.7)	133 (18.2)	<0.0001
Midwest	117 (15.6)	240 (20.5)		176 (14.8)	181 (24.8)	
South	140 (18.6)	238 (20.4)		263 (22.1)	115 (15.8)	
West	321 (42.7)	462 (39.5)		482 (40.5)	301 (41.2)	
*Hospital esophagectomy volume*						
Quintile 1: 1–9	191 (25.4)	161 (13.8)	<0.0001	230 (19.3)	122 (16.7)	<0.0001
Quintile 2: 10–22	182 (24.2)	233 (19.9)		279 (23.4)	136 (18.6)	
Quintile 3: 23–49	143 (19.0)	231 (19.8)		250 (21.0)	124 (17.0)	
Quintile 4: 50–87	114 (15.2)	275 (23.5)		255 (21.4)	134 (18.4)	
Quintile 5: 88–209	122 (16.2)	269 (23.0)		177 (14.9)	214 (29.3)	
*Education*^†^						
Q1: 2.5%-<13.3%	175 (23.3)	265 (22.8)	0.0355	289 (24.3)	151 (20.7)	0.1655
Q2: 13.3%-<18.4%	166 (22.1)	322 (27.5)		309 (25.9)	179 (24.5)	
Q3: 18.5%-<23.0%	205 (27.3)	309 (26.4)		307 (25.8)	207 (28.4)	
Q4: 23.1%-<45.7%	206 (27.4)	273 (23.4)		286 (24.0)	193 (26.4)	
*Income*^‡^						
Q1: $25,717-$46,451	175 (23.3)	263 (22.5)	0.0460	297 (24.9)	141 (19.3)	0.0002
Q2: $46,452-$53,263	218 (29.0)	324 (27.7)		313 (26.3)	229 (31.4)	
Q3: $53,264-$62,815	144 (19.2)	286 (24.5)		288 (24.2)	142 (19.5)	
Q4: $62,816-$91,050	215 (28.6)	296 (25.3)		293 (24.6)	218 (29.9)	

SD, Standard Deviation

TTE, Transthoracic esophagectomy

THE, Transhiatal esophagectomy

Q, Quartile

Data are presented as frequency (%) unless otherwise specified

^†^Mean % residents in county with college education

^‡^Mean county-level median income

### Short-term outcomes

Patients with EA had lower 30-day and 90-day mortality than those without. Per NCI policy, 30-day mortality rates cannot be reported due to counts <11. Ninety-day mortality was 5.6% (65/1,169) with EA and 8.9% (67/752) without EA (p = 0.005).

### Overall survival

**Fig 2:**
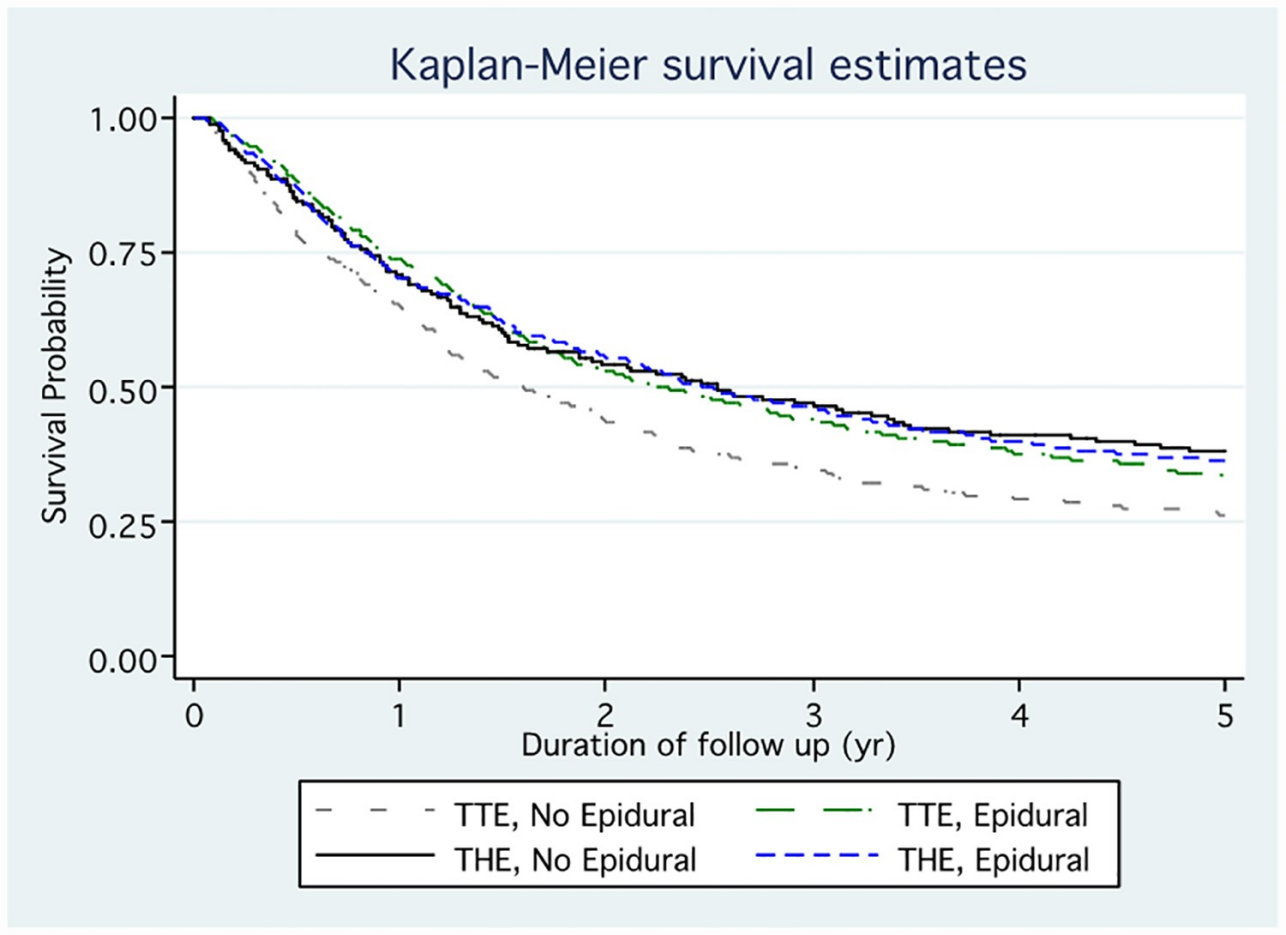
Kaplan-Meier overall survival curves for patients undergoing transthoracic or transhiatal esophagectomy with or without epidural analgesia.

**Fig 3:**
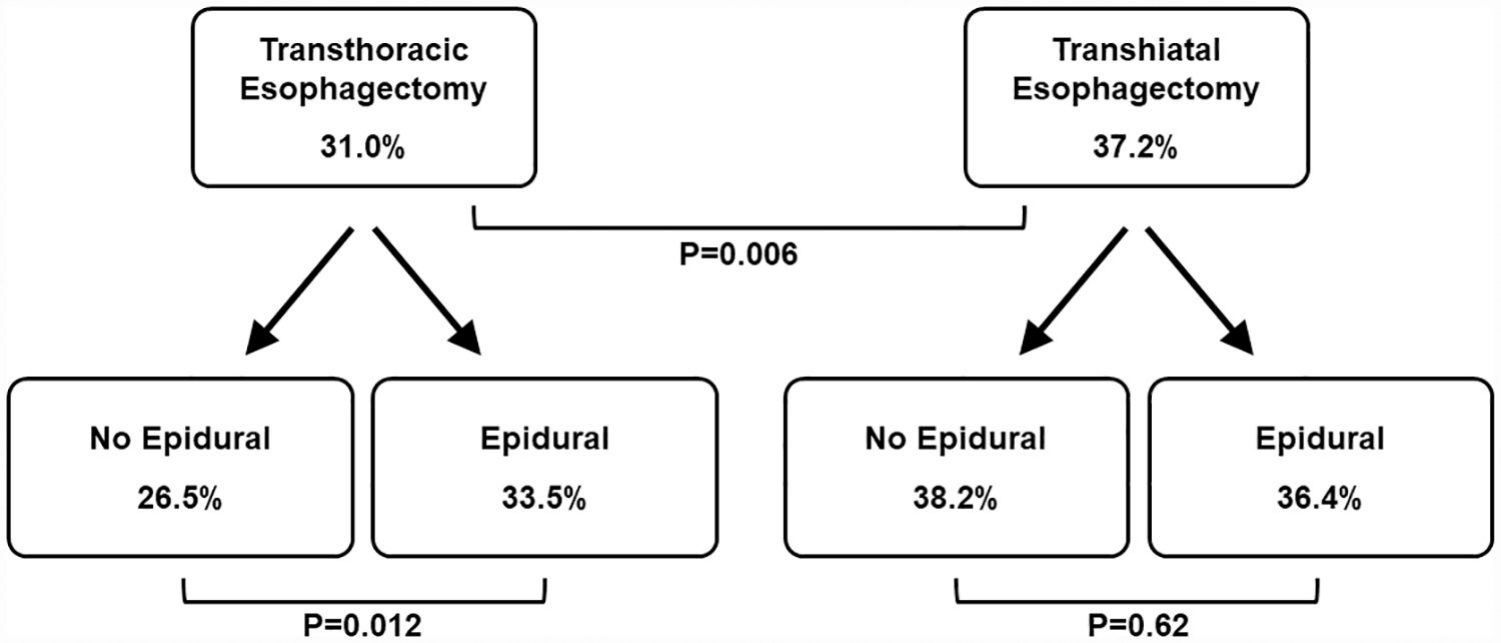
Five-year survival by surgical approach and, within each surgical group, by epidural status. P values are from Z-tests comparing Kaplan-Meier estimates of 5-year survival rates.

Fig 2 shows Kaplan-Meier curves displaying survival by pain management and surgical approaches. Five-year survival was better with THE versus TTE and, among TTE patients, better with EA (Fig 3). The multivariable marginal Cox model examining the association between EA and time to death is shown (Table 2), including an interaction term for surgical approach and epidural status which was significant (p = 0.009). The adjusted mortality hazard ratio (HR) for EA among TTE patients was 0.81 (95% confidence interval [CI], 0.70, 0.93; p = 0.0035), indicating better survival among TTE patients receiving EA. The HR for EA among THE patients was non-significant (1.10, 95% CI 0.91, 1.34).

**Table 2:** Marginal Cox model for time to death (5-year survival).

Variable	HR (95%CI)	P value
*Epidural receipt*^†^		
Among those receiving TTE	0.81 (0.70–0.93)	0.0035
Among those receiving THE	1.10 (0.91–1.34)	0.3023
*Transhiatal esophagectomy*^†^		
Among those receiving epidural	1.03 (0.88–1.21)	0.6924
Among those not receiving epidural	0.75 (0.63–0.90)	0.002
Age at diagnosis	1.04 (1.03–1.05)	<0.0001
Female gender vs. male	0.83 (0.72–0.96)	0.0099
Black/Other race vs. white	0.98 (0.79–1.20)	0.8150
Adenocarcinoma vs. SCC	0.76 (0.67–0.86)	<0.0001
Regional stage vs. localized	2.14 (1.90–2.42)	<0.0001
*Charlson comorbidity score*		
0	Reference	
1	1.14 (1.01–1.29)	0.0339
≥2	1.41 (1.10–1.80)	0.0068
Perioperative transfusion	1.02 (0.86–1.22)	0.8041
Radiation	0.92 (0.82–1.04)	0.1789
*SEER registry region*		
Northeast	Reference	
Midwest	1.13 (0.93–1.39)	0.2247
South	0.99 (0.81–1.21)	0.9225
West	0.93 (0.79–1.09)	0.3797
*Hospital esophagectomy volume*		
Quintile 1: 1–9	Reference	
Quintile 2: 10–22	0.81 (0.69–0.96)	0.0152
Quintile 3: 23–49	0.72 (0.60–0.85)	0.0002
Quintile 4: 50–87	0.62 (0.52–0.75)	<0.0001
Quintile 5: 88–209	0.52 (0.43–0.63)	<0.0001
*Education*^‡^		
Q1: 2.5%-13.3%	1.28 (0.98–1.67)	0.0674
Q2: 13.4%-18.4%	1.10 (0.88–1.37)	0.4103
Q3: 18.5%-22.9%	1.25 (1.02–1.54)	0.0340
Q4: 23.0%-45.7%	Reference	
*Income*^§^		
Q1: $25,717-$46,451	0.97 (0.75–1.27)	0.8397
Q2: $46,452-$53,263	1.00 (0.80–1.25)	0.9738
Q3: $53,264-$62,815	1.03 (0.84–1.27)	0.7585
Q4: $62,816-$91,050	Reference	

TTE, Transthoracic esophagectomy

THE, Transhiatal esophagectomy

SCC, Squamous cell carcinoma

Q, Quartile

^†^Test for the interaction between surgical approach and epidural status was significant (p = 0.0092)

^‡^Mean % residents in county with college education

^§^Mean county-level median income

Surgical approach also impacted survival: among non-epidural patients, THE was associated with lower mortality (HR 0.75, 95% CI 0.63, 0.90), while there was no association with mortality among THE patients receiving EA (HR 1.03, 95% CI 0.88, 1.21). Female gender, adenocarcinoma, and higher hospital volume were associated with a decreased risk of death, while age, regional stage, and Charlson score of 1 or ≥2 were predictors of mortality. Radiation in the first course of treatment and perioperative transfusions were not significantly associated with time to death. Sensitivity analyses examining the impact of epidural receipt on OS within each surgical group also demonstrated an association between EA and decreased mortality with TTE (HR 0.81, 95% CI 0.70, 0.93; S1 Table), but not with THE (HR 1.09, 95% CI 0.89, 1.33). Additionally, in sensitivity analyses stratified by propensity score quartile within each surgical group, EA was associated with decreased mortality with TTE (HR 0.79, 95% CI 0.68, 0.92; S2 Table), but not with THE (HR 1.09, 95% CI 0.90, 1.33; S2 Table).

### Time to recurrence

The recurrence analysis included 1,635 patients (85% of the cohort) surviving ≥6 months, among whom 62.5% (n = 1,023) received epidurals and 37.4% (n = 612) did not. Fifty-three percent of these patients (n = 864) met criteria for recurrence. In a multivariable marginal Cox model, hazard ratios for the association between EA and time to recurrence were 0.86 (95% CI 0.72, 1.04) for TTE and 1.14 (95% CI 0.90, 1.43) for THE (Table 3). To maintain consistency with the OS model, an interaction term between surgical approach and epidural receipt was included although the term was not significant (p = 0.069). Adenocarcinoma was associated with decreased recurrence, while increasing age and regional stage were associated with increased risk of recurrence. Marginal Cox models within each surgical group did not show an association between EA and time to recurrence (TTE, 0.85 [95% CI 0.70, 1.02]; THE, 1.09 [95% CI 0.85, 1.38]; S1 Table).

**Table 3:** Marginal Cox model for time to recurrence.

Variable	HR (95%CI)	P value
*Epidural receipt*^†^		
Among those receiving TTE	0.86 (0.72–1.04)	0.1188
Among those receiving THE	1.14 (0.90–1.43)	0.2833
*Transhiatal esophagectomy*^†^		
Among those receiving epidural	1.06 (0.87–1.28)	0.5792
Among those not receiving epidural	0.80 (0.64–1.01)	0.0589
Age at diagnosis	1.02 (1.00–1.03)	0.0111
Female gender vs. male	0.91 (0.77–1.09)	0.3049
Black/Other race vs. white	1.11 (0.87–1.42)	0.4172
Adenocarcinoma vs. SCC	0.79 (0.67–0.92)	0.0032
Regional stage vs. localized	2.34 (2.00–2.73)	<0.0001
*Charlson comorbidity score*		
0	Reference	
1	1.10 (0.95–1.28)	0.2032
≥2	1.33 (0.98–1.81)	0.0716
Perioperative transfusion	0.92 (0.73–1.15)	0.4466
Radiation	1.33 (1.14–1.54)	0.0003
*SEER registry region*		
Northeast	Reference	
Midwest	0.90 (0.70–1.16)	0.4261
South	0.98 (0.76–1.25)	0.8439
West	0.89 (0.73–1.09)	0.2527
*Hospital esophagectomy volume*		
Quintile 1: 1–10	Reference	
Quintile 2: 11–24	0.84 (0.68–1.04)	0.1133
Quintile 3: 25–52	0.84 (0.67–1.04)	0.1085
Quintile 4: 53–94	0.74 (0.59–0.92)	0.0075
Quintile 5: 95–209	0.76 (0.60–0.96)	0.0223
*Education*^‡^		
Q1: 2.5%-13.3%	1.20 (0.86–1.69)	0.2888
Q2: 13.4%-18.4%	1.19 (0.90–1.57)	0.2245
Q3: 18.5%-23.0%	1.19 (0.91–1.55)	0.1962
Q4: 23.1%-45.7%	Reference	
*Income*^§^		
Q1: $24,869-$46,451	0.97 (0.69–1.36)	0.8522
Q2: $46,452-$52,489	0.95 (0.72–1.27)	0.7442
Q3: $52,490-$62,815	1.09 (0.84–1.41)	0.5360
Q4: $62,816-$91,050	Reference	

TTE, Transthoracic esophagectomy

THE, Transhiatal esophagectomy

SCC, Squamous cell carcinoma

Q, Quartile

^†^Test for the interaction between surgical approach and epidural status was not significant (p = 0.069)

^‡^Mean % residents in county with college education

^§^Mean county-level median income

### Subgroup analysis restricted to esophageal adenocarcinoma

A subgroup analysis restricted to the 1,343 esophageal adenocarcinoma patients in the cohort was performed. Among adenocarcinoma patients, 817 underwent TTE and 526 underwent THE (Table 4). EA was utilized in 61% of cases (n = 821), remained associated with higher esophagectomy volume (p<0.0001), and was more common with TTE (64.5%, n = 527 vs. 55.9%, n = 294 for THE). A marginal Cox model assessing the impact of EA on long-term survival demonstrated that EA was associated with improved survival among adenocarcinoma patients receiving TTE (Table 5; HR 0.81, 95% CI 0.67, 0.96) but not among adenocarcinoma patients receiving THE (Table 5; HR 1.01, 95% CI 0.80, 1.27), while adjusting for other factors. There was no significant association between EA and time to recurrence among adenocarcinoma patients receiving TTE (HR 0.87, 95% CI 0.70, 1.10) or THE (HR 1.11, 95% CI 0.83, 1.46). In sensitivity analyses stratified by PS quartile, among patients undergoing TTE, EA was associated with improved survival (S3 Table; HR 0.80, 95% CI 0.67, 0.96) but not improved time to recurrence (HR 0.88, 95% CI 0.70, 1.10).

**Table 4:** Baseline characteristics among adenocarcinoma patients by pain management approach and surgical approach.

	Pain management approach			Surgical approach		
	No Epidural (n = 522)	Epidural (n = 821)	P value	TTE (n = 817)	THE (n = 526)	P value
Mean age (SD)	74.1 (5.1)	73.3 (4.9)	0.0031	73.4 (4.8)	73.9 (5.1)	0.0813
Female	76 (14.6)	114 (13.9)	0.7298	107 (13.1)	83 (15.8)	0.1685
Black/Other race	23 (4.4)	19 (2.3)	0.0318	24 (2.9)	18 (3.4)	0.6185
Localized stage	243 (46.6)	356 (43.4)	0.2516	356 (43.6)	243 (46.2)	0.3451
*Charlson comorbidity score*						
0	162 (31.0)	255 (32.1)	0.5375	266 (32.6)	151 (28.7)	0.0115
1	328 (62.8)	527 (64.2)		519 (63.5)	336 (63.9)	
≥2	32 (6.1)	39 (4.8)		32 (3.9)	39 (7.4)	
TTE	290 (55.6)	527 (64.2)	0.0016			
Epidural				527 (64.5)	294 (55.9)	0.0016
Perioperative transfusion	55 (10.5)	86 (10.5)	0.9715	93 (11.4)	48 (9.1)	0.1877
Radiation	202 (38.7)	348 (42.4)	0.1801	332 (40.6)	218 (41.4)	0.7687
*SEER registry region*						
Northeast	117 (22.4)	163 (19.9)	0.2540	181 (22.2)	99 (18.8)	<0.0001
Midwest	87 (16.7)	171 (20.8)		126 (15.4)	132 (25.1)	
South	102 (19.5)	160 (19.5)		175 (21.4)	87 (16.5)	
West	216 (41.4)	327 (39.8)		335 (41.0)	208 (39.5)	
*Hospital esophagectomy volume*						
Quintile 1: 1–9	131 (25.1)	111 (13.5)	<0.0001	168 (20.6)	74 (14.1)	<0.0001
Quintile 2: 10–22	122 (23.4)	163 (19.9)		184 (22.5)	101 (19.2)	
Quintile 3: 23–49	101 (19.0)	170 (20.7)		183 (22.4)	88 (16.7)	
Quintile 4: 50–87	87 (16.7)	183 (22.3)		164 (20.1)	106 (20.1)	
Quintile 5: 88–209	81 (15.5)	194 (23.6)		118 (14.4)	157 (29.9)	
*Education*^†^						
Q1: 2.5%-<13.4%	133 (25.5)	198 (24.1)	0.3860	216 (26.4)	115 (21.9)	0.2455
Q2: 13.4%-<18.4%	116 (22.2)	217 (26.4)		201 (24.6)	132 (25.1)	
Q3: 18.4%-<22.5%	127 (24.3)	190 (23.1)		183 (22.4)	134 (25.5)	
Q4: 22.5%-<39.2%	146 (28.0)	216 (26.3)		217 (26.6)	145 (27.6)	
*Income*^‡^						
Q1: $25,717-$46,451	137 (26.3)	192 (23.4)	0.0261	219 (26.8)	110 (20.9)	0.0101
Q2: $46,452-$52,097	140 (26.8)	190 (23.1)		186 (22.8)	144 (27.4)	
Q3: $52,098-$62,815	108 (20.7)	228 (27.8)		215 (26.3)	121 (23.0)	
Q4: $62,816-$91,050	137 (26.3)	211 (25.7)		197 (24.1)	151 (28.7)	

SD, Standard Deviation

TTE, Transthoracic esophagectomy

THE, Transhiatal esophagectomy

Q, Quartile

Data are presented as frequency (%) unless otherwise specified

^†^Mean % residents in county with college education

^‡^Mean county-level median income

**Table 5:** Marginal Cox model for time to death (5-year survival) in adenocarcinoma patients.

Variable	HR (95%CI)	P value
*Epidural receipt*^†^		
Among those receiving TTE	0.81 (0.67–0.96)	0.0174
Among those receiving THE	1.01 (0.80–1.27)	0.9530
*Transhiatal esophagectomy*^†^		
Among those receiving epidural	0.92 (0.76–1.11)	0.3738
Among those not receiving epidural	0.74 (0.59–0.92)	0.0061
Age at diagnosis	1.05 (1.03–1.06)	<0.0001
Female gender vs. male	0.73 (0.59–0.90)	0.0032
Black/Other race vs. white	0.81 (0.55–1.21)	0.8100
Regional stage vs. localized	2.33 (2.00–2.71)	<0.0001
*Charlson comorbidity score*		
0	Reference	
1	1.12 (0.96–1.30)	0.1565
≥2	1.60 (1.18–2.19)	0.0027
Perioperative transfusion	1.06 (0.85–1.31)	0.6190
Radiation	0.97 (0.84–1.13)	0.6848
*SEER registry region*		
Northeast	Reference	
Midwest	1.12 (0.87–1.43)	0.3896
South	0.97 (0.76–1.23)	0.7967
West	0.91 (0.75–1.14)	0.3662
*Hospital esophagectomy volume*		
Quintile 1: 1–9	Reference	
Quintile 2: 10–22	0.85 (0.69–1.05)	0.1292
Quintile 3: 23–49	0.70 (0.57–0.87)	0.0002
Quintile 4: 50–87	0.60 (0.52–0.75)	<0.0001
Quintile 5: 88–209	0.53 (0.42–0.67)	<0.0001
*Education*^‡^		
Q1: 2.5%-<13.4%	1.19 (0.86–1.64)	0.3038
Q2: 13.4%-<18.4%	1.08 (0.81–1.42)	0.6120
Q3: 18.4%-<22.7%	1.25 (0.96–1.62)	0.1006
Q4: 22.7%-39.2%	Reference	
*Income*^§^		
Q1: $25,717-$46,451	1.01 (0.73–1.39)	0.9660
Q2: $46,452-$52,446	1.02 (0.78–1.35)	0.8734
Q3: $52,447-$62,815	1.14 (0.87–1.48)	0.3449
Q4: $62,816-$91,050	Reference	

TTE, Transthoracic esophagectomy; THE, Transhiatal esophagectomy

SCC, Squamous cell carcinoma

Q, Quartile

^†^Test for interaction of surgical approach with epidural status was not significant (p = 0.1517)

^‡^Mean % residents in county with college education

^§^Mean county-level median income

## Discussion

To our knowledge, this is the first population-based analysis of analgesic technique and long-term outcomes after esophagectomy. We found an interaction between epidural analgesia and surgical approach in the survival analysis; i.e., the effects of epidural analgesia and surgical approach on overall survival were dependent upon each other. Among non-epidural patients, THE was associated with improved overall survival. Among patients undergoing TTE, EA was associated with improved survival, including the subgroup of adenocarcinoma patients. This latter finding is consistent with our prior work in colorectal cancer[21] and a recent meta-analysis of the effects of EA on multiple outcomes.[30] Our results are also supported by a recent meta-analysis demonstrating improved overall and recurrence-free survival in patients receiving neuraxial anesthesia and/or analgesia for cancer resection.[31]

In the current study, EA was associated with higher hospital esophagectomy volume, which has been linked to improved survival.[12, 13, 32] We found that higher hospital esophagectomy volumes were independently associated with better survival (Table 2). Patients receiving EA were younger. Adenocarcinoma was associated with better survival, consistent with prior studies,[33, 34] while increasing age, higher comorbidity, and regional stage were associated with worse survival.

Improved survival with EA among TTE patients in our study could result from fewer postoperative complications. Prior studies have demonstrated beneficial effects of EA on numerous outcomes including anastomotic leak and respiratory failure.[30, 35, 36] Epidural analgesia could be associated with improved survival because higher-volume centers providing EA would have infrastructure and specialists needed to optimize management of major complications. Surgical outcomes, including the ability to rescue patients from major complications, have been linked to the degree of anesthesiologist involvement.[37] Additionally, although our analysis adjusts for Charlson score and age, patients receiving EA may have been healthier as a result of unmeasured comorbidities.

Although EA was associated with better survival within the TTE group, we found no association with time to recurrence. The recurrence algorithm, although intended to be specific, likely lacks sensitivity for non-treated recurrence and misclassifies some untreated recurrences as non-recurrence. This underscores the difficulty defining cancer recurrence using claims data without access to medical records.[38] Alternatively, there may be no effect of epidural analgesia on recurrence and any survival benefit would result from reduced perioperative complications.

In contrast with our previous studies,[21, 24] this analysis did not demonstrate any difference in survival with blood transfusions. Transfusions were likely undercoded, since other studies have reported transfusion rates of 38%-40% with esophagectomy.[39, 40] Despite prospective data in colorectal cancer showing that transfusion is associated with increased risk of recurrence,[17] the low transfusion rates of 9%-12% seen in this study reduced statistical power to detect an association with recurrence.

THE was associated with improved survival among non-epidural patients, adjusting for other factors including comorbid conditions, histology, and stage. This association remained in the subgroup analysis restricted to adenocarcinoma cases (Table 5). Previous analyses comparing TTE to THE have not included EA as a covariate. Chang et al. demonstrated higher 5-year survival for THE than TTE (30.5% vs. 22.7%, p = 0.02), but this difference did not persist after adjustment.[11] Other studies found no survival difference by surgical approach.[41, 42] A randomized trial showed potential benefit for TTE among patients with 1–8 positive nodes.[43] Some analyses report greater lymph node yield with TTE.[44, 45] Our findings may result from selection bias. The THE group was more likely to have localized stage; although our analysis adjusted for stage, confounders such as margin positivity, unmeasured comorbidities, or provider preferences (i.e., reserving TTE for more aggressive disease or younger patients) could have biased the results.

This analysis has several other limitations. Given its observational nature, causality cannot be inferred. Untreated recurrence could have been misclassified. Because detailed staging information is often incomplete, our analysis used SEER stage, which is more consistent over time although lacking granularity. We were also unable to distinguish open and minimally-invasive approaches. This would minimize differences between groups as patients undergoing minimally-invasive esophagectomy are less likely to receive EA. Over the study period, however, minimally-invasive approaches were less common than at present. To allow adequate follow-up, the cohort was from 2009 and earlier. Finally, clinical data regarding analgesic approaches in the non-epidural group were not available, resulting in a heterogenous group. Likewise, in the epidural group, clinical data detailing the degree of success of the epidural block were not available. However, patients with ineffective epidural analgesia would have been included in the EA group, which would have minimized the observed differences.

Our study has several strengths including a large sample size for a relatively uncommon malignancy. Our results are potentially more generalizable to the elderly than clinical trials, which often do not include older patients.[46] While a randomized trial comparing operative and analgesic approaches provides the strongest study design, physician biases and patient preferences could hamper accrual, as occurred with CALGB 9781.[47] Nonetheless, an ongoing study comparing epidural analgesia to intravenous patient-controlled analgesia for pancreatoduodenectomy demonstrates that randomized controlled trials comparing alternative approaches in acute pain management are possible.[48]

## Conclusions

In summary, this large population-based analysis demonstrates an association between epidural analgesia and improved survival after TTE, but not THE, for cancer. This association persisted in analyses restricted to patients with adenocarcinoma. Since no association between EA and recurrence was found, the results do not support the hypothesis that EA protects against recurrence by reducing immunosuppression and improving tumor surveillance when compared to systemic opioids. Given the limitations of SEER-Medicare data, prospective studies or registries with more detailed clinical information are needed to elucidate the effect of regional analgesic techniques on esophageal cancer recurrence, particularly in the era of minimally invasive approaches. Nonetheless, our findings support the importance of appropriate analgesic selection tailored to the surgical approach for esophageal cancer.

## Supporting information

S1 File Appendix. (DOCX)

S2 File STROBE checklist. (DOC)

S1 Table Marginal Cox models for time to death and time to recurrence within TTE group. (DOCX)

S2 Table Marginal Cox model for time to death (5-year survival) within each surgical group—Stratified by propensity score quartiles. (DOCX)

S3 Table Marginal Cox model for time to death (5-year survival) in adenocarcinoma patients—Stratified by propensity score quartiles. (DOCX)
