# Correction: Surgical approach and the impact of epidural analgesia on survival after esophagectomy for cancer: A population-based retrospective cohort study

**DOI:** 10.1371/journal.pone.0214528

**Published:** 2019-03-21

**Authors:** The PLOS ONE Staff

The following information is missing from the Funding statement: Publication costs for the article were supported by a Case Western Reserve University VPR Catalyst Award to LCC.

The publisher apologizes for this error.
