# Supplementary material for: Surgical approach and the impact of epidural analgesia on survival after esophagectomy for cancer: A population-based retrospective cohort study
Source: PLoS One. 2019 Jan 22;14(1):e0211125. doi: 10.1371/journal.pone.0211125 (PMC6342325; doi:10.1371/journal.pone.0211125)
Supplement: S1 File — (DOCX) [file pone.0211125.s001.docx]

**Appendix**

**Codes used to identify esophagectomy**

Transhiatal esophagectomy: CPT codes 43107, 43119

Transthoracic esophagectomy: CPT codes 43112, 43117, 43121, 43122

**Secondary neoplasm ICD-9-CM diagnosis codes**

197.0, 197.1, 197.2, 197.3, 197.6, 197.7, 198.3, 198.4, 198.5, 789.51
