# Supplementary material for: Surgical approach and the impact of epidural analgesia on survival after esophagectomy for cancer: A population-based retrospective cohort study
Source: PLoS One. 2019 Jan 22;14(1):e0211125. doi: 10.1371/journal.pone.0211125 (PMC6342325; doi:10.1371/journal.pone.0211125)
Supplement: S2 File — (DOC) [file pone.0211125.s002.doc]

STROBE Statement—Checklist of items that should be included in reports of ***cohort studies***

|  | Item No | Recommendation |
| --- | --- | --- |
| **Title and abstract** | 1 | (*a*) Indicate the study’s design with a commonly used term in the title or the abstract |
|  |  | The cohort study design is indicated in the methods section of the abstract.  (*b*) Provide in the abstract an informative and balanced summary of what was done and what was found  Within the limitations of the word count, the abstract summarizes the key methodology and findings of the study. |
| Introduction | | |
| Background/rationale | 2 | Explain the scientific background and rationale for the investigation being reported |
|  |  | The scientific background and rationale are detailed in the Introduction section. |
| Objectives | 3 | State specific objectives, including any prespecified hypotheses |
|  |  | The aims/prespecified hypotheses are listed in the 3rd paragraph of the introduction section. |
| Methods | | |
| Study design | 4 | Present key elements of study design early in the paper |
|  |  | Key elements of the study design are provided in the 1st paragraph of the materials and methods section. |
| Setting | 5 | Describe the setting, locations, and relevant dates, including periods of recruitment, exposure, follow-up, and data collection |
|  |  | See Materials and Methods (Data Sources, Study population, Measures, and Statistical Analysis sections). |
| Participants | 6 | (*a*) Give the eligibility criteria, and the sources and methods of selection of participants. Describe methods of follow-up |
|  |  | (*b*) For matched studies, give matching criteria and number of exposed and unexposed |
|  |  | See Materials and Methods, Data Sources and Study Population sections. |
| Variables | 7 | Clearly define all outcomes, exposures, predictors, potential confounders, and effect modifiers. Give diagnostic criteria, if applicable |
|  |  | See Materials and Methods, Measures section. The Appendix provides procedure codes used to identify treatment approach. |
| Data sources/ measurement | 8* | For each variable of interest, give sources of data and details of methods of assessment (measurement). Describe comparability of assessment methods if there is more than one group |
|  |  | See Materials and Methods, Measures section and Appendix for details regarding variables of interest and methods of assessment for treatment groups. |
| Bias | 9 | Describe any efforts to address potential sources of bias |
|  |  | See Materials and Methods, Statistical analysis section, regarding adjustment for hospital clustering and an interaction between surgical approach and epidural status. This section also describes a subgroup analysis restricted to esophageal adenocarcinoma patients. |
| Study size | 10 | Explain how the study size was arrived at |
|  |  | See Results, 1st paragraph and Figure 1 regarding numbers of patients excluded. |
| Quantitative variables | 11 | Explain how quantitative variables were handled in the analyses. If applicable, describe which groupings were chosen and why |
|  |  | See Materials and Methods, Measures section, Esophagectomy and hospital esophagectomy volume subsection and Materials and Methods, Statistical analysis section. |
| Statistical methods | 12 | (*a*) Describe all statistical methods, including those used to control for confounding |
|  |  | (*b*) Describe any methods used to examine subgroups and interactions |
|  |  | (*c*) Explain how missing data were addressed |
|  |  | (*d*) If applicable, explain how loss to follow-up was addressed |
|  |  | (*e*) Describe any sensitivity analyses |
|  |  | See Materials and Methods, Statistical Analysis section. See Materials and Methods, Measures section, Esophagectomy and hospital esophagectomy volume subsection regarding missing hospital identifiers. |
| Results | | |
| Participants | 13* | (a) Report numbers of individuals at each stage of study—eg numbers potentially eligible, examined for eligibility, confirmed eligible, included in the study, completing follow-up, and analysed |
|  |  | (b) Give reasons for non-participation at each stage |
|  |  | (c) Consider use of a flow diagram |
|  |  | See Figure 1 for flow diagram regarding numbers of patients excluded and reasons for exclusion. |
| Descriptive data | 14* | (a) Give characteristics of study participants (eg demographic, clinical, social) and information on exposures and potential confounders |
|  |  | (b) Indicate number of participants with missing data for each variable of interest |
|  |  | (c) Summarise follow-up time (eg, average and total amount) |
|  |  | See Table 1 for baseline characteristics of study patients. See Figure 1 for patients excluded for missing data. See Results, 1^st^ paragraph (last 2 sentences) regarding follow-up time. |
| Outcome data | 15* | Report numbers of outcome events or summary measures over time |
|  |  | See Results, Short-term outcomes section and Overall survival section. |
| Main results | 16 | (*a*) Give unadjusted estimates and, if applicable, confounder-adjusted estimates and their precision (eg, 95% confidence interval). Make clear which confounders were adjusted for and why they were included |
|  |  | (*b*) Report category boundaries when continuous variables were categorized |
|  |  | (*c*) If relevant, consider translating estimates of relative risk into absolute risk for a meaningful time period |
|  |  | Hazard ratios and associated 95% confidence intervals are presented in the survival and recurrence analyses (Tables 2, 3, and 5). Cutoffs for categorized continuous variables (geographic characteristics and hospital esophagectomy volume) are provided in the tables. |
| Other analyses | 17 | Report other analyses done—eg analyses of subgroups and interactions, and sensitivity analyses |
|  |  | Tables 2 and 3 include an interaction term for surgical approach and epidural status. Tables 4 and 5 report results of a subgroup analysis restricted to adenocarcinoma patients. Supplementary Table 1 reports results for survival and recurrence analyses within the transthoracic esophagectomy group. |
| Discussion | | |
| Key results | 18 | Summarise key results with reference to study objectives |
|  |  | See Discussion, 1^st^ paragraph. |
| Limitations | 19 | Discuss limitations of the study, taking into account sources of potential bias or imprecision. Discuss both direction and magnitude of any potential bias |
|  |  | See Discussion, 7^th^ paragraph for limitations. See Discussion, 3^rd^ and 6^th^ paragraphs for consideration of bias. |
| Interpretation | 20 | Give a cautious overall interpretation of results considering objectives, limitations, multiplicity of analyses, results from similar studies, and other relevant evidence |
|  |  | See Discussion. |
| Generalisability | 21 | Discuss the generalisability (external validity) of the study results |
|  |  | See Discussion, 8^th^ paragraph. |
| Other information | | |
| Funding | 22 | Give the source of funding and the role of the funders for the present study and, if applicable, for the original study on which the present article is based |
|  |  | See Funding Statement. |

*Give information separately for exposed and unexposed groups.

**Note:** An Explanation and Elaboration article discusses each checklist item and gives methodological background and published examples of transparent reporting. The STROBE checklist is best used in conjunction with this article (freely available on the Web sites of PLoS Medicine at http://www.plosmedicine.org/, Annals of Internal Medicine at http://www.annals.org/, and Epidemiology at http://www.epidem.com/). Information on the STROBE Initiative is available at http://www.strobe-statement.org.
