# Supplementary material for: Surgical approach and the impact of epidural analgesia on survival after esophagectomy for cancer: A population-based retrospective cohort study
Source: PLoS One. 2019 Jan 22;14(1):e0211125. doi: 10.1371/journal.pone.0211125 (PMC6342325; doi:10.1371/journal.pone.0211125)
Supplement: S1 Table — (DOCX) [file pone.0211125.s003.docx]

**S1 Table.** Marginal Cox models for time to death and time to recurrence within TTE group

|  | **Time to death**  **(n= 1,191)** | | **Time to recurrence (n=1,009)** | |
| --- | --- | --- | --- | --- |
| **Variable** | **HR (95%CI)** | **P value** | **HR (95%CI)** | **P value** |
| Epidural vs. no epidural | 0.81 (0.70-0.93) | 0.0036 | 0.85 (0.70-1.02) | 0.0800 |
| Age at diagnosis | 1.05 (1.03-1.06) | <0.0001 | 1.02 (1.01-1.04) | 0.0148 |
| Female gender vs. male | 0.83 (0.69-0.99) | 0.0417 | 0.96 (0.77-1.19) | 0.6918 |
| Black/Other race vs. white | 0.96 (0.74-1.23) | 0.7235 | 1.15 (0.85-1.55) | 0.3815 |
| Adenocarcinoma vs. SCC | 0.83 (0.71-0.98) | 0.0235 | 0.87 (0.71-1.07) | 0.1753 |
| Regional stage vs. localized | 2.11 (1.81-2.45) | <0.0001 | 2.14 (1.77-2.59) | <0.0001 |
| *Charlson comorbidity score* | | | | |
| 0 | Reference |  | Reference |  |
| 1 | 1.02 (0.88-1.18) | 0.8263 | 1.04 (0.87-1.26) | 0.6480 |
| ≥2 | 1.46 (1.05-2.01) | 0.0232 | 1.28 (0.83-1.96) | 0.2629 |
| Perioperative transfusion | 0.92 (0.74-1.15) | 0.4833 | 0.84 (0.63-1.10) | 0.2063 |
| Radiation | 0.99 (0.85-1.15) | 0.8581 | 1.37 (1.14-1.65) | 0.0009 |
| *SEER registry region* | | | | |
| Northeast | Reference |  | Reference |  |
| Midwest | 1.03 (0.79-1.33) | 0.8459 | 0.81 (0.58-1.12) | 0.2043 |
| South | 1.02 (0.80-1.30) | 0.8863 | 1.05 (0.79-1.42) | 0.7253 |
| West | 0.89 (0.73-1.08) | 0.2367 | 0.85 (0.66-1.08) | 0.1865 |
| *Hospital esophagectomy volume*^†^ | | | | |
| Quintile 1: 0-19^th^ %ile | Reference |  | Reference |  |
| Quintile 2: 20th-39^th^ %ile | 0.90 (0.73-1.11) | 0.3371 | 0.96 (0.73-1.26) | 0.7719 |
| Quintile 3: 40^th^-59^th^ %ile | 0.74 (0.60-0.92) | 0.0069 | 0.93 (0.70-1.23) | 0.6023 |
| Quintile 4: 60^th^-79^th^ %ile | 0.74 (0.59-0.93) | 0.0097 | 0.88 (0.66-1.17) | 0.3721 |
| Quintile 5: 80^th^-99^th^ %ile | 0.55 (0.43-0.69) | <0.0001 | 0.76 (0.57-1.00) | 0.0489 |
| *Education*^‡^ | | | | |
| Q1: 2.5%-13.3% | 1.23 (0.89-1.69) | 0.2183 | 1.11 (0.73-1.67) | 0.6290 |
| Q2: 13.4%-18.4% | 1.17 (0.89-1.53) | 0.2636 | 1.32 (0.94-1.84) | 0.1043 |
| Q3: 18.5%-23.0% | 1.36 (1.07-1.74) | 0.0132 | 1.31 (0.96-1.78) | 0.0846 |
| Q4: 23.1%-45.7% | Reference |  | Reference |  |
| *Income*^§^ | | | | |
| Q1: $25,717-$46,451 | 0.97 (0.71-1.34) | 0.8728 | 0.94 (0.63-1.41) | 0.7721 |
| Q2: $46,452-$53,263 | 0.96 (0.73-1.26) | 0.7753 | 0.86 (0.61-1.22) | 0.4023 |
| Q3: $53,264-$62,815 | 1.00 (0.78-1.28) | 0.9781 | 0.98 (0.73-1.32) | 0.8979 |
| Q4: $62,816-$91,050 | Reference |  | Reference |  |

TTE, Transthoracic esophagectomy

SCC, Squamous cell carcinoma

Q, Quartile

^†^Quintiles for esophagectomy volume were calculated using cutoffs for the TTE group

^‡^Mean % residents in county with college education

^§^Mean county-level median income
