# Supplementary material for: Surgical approach and the impact of epidural analgesia on survival after esophagectomy for cancer: A population-based retrospective cohort study
Source: PLoS One. 2019 Jan 22;14(1):e0211125. doi: 10.1371/journal.pone.0211125 (PMC6342325; doi:10.1371/journal.pone.0211125)
Supplement: S2 Table — (DOCX) [file pone.0211125.s004.docx]

**S2 Table.** Marginal Cox models for time to death (5-year survival) within each surgical group - stratified by propensity score quartiles

|  | **TTE (n= 1,191)** | | **THE (n=730)** | |
| --- | --- | --- | --- | --- |
| **Variable** | **HR (95%CI)** | **P value** | **HR (95%CI)** | **P value** |
| Epidural vs. no epidural | 0.79 (0.68-0.92) | 0.0020 | 1.09 (0.90-1.33) | 0.3778 |
| Age at diagnosis | 1.05 (1.04-1.07) | <0.0001 | 1.02 (1.00-1.04) | 0.0341 |
| Female gender vs. male | 0.83 (0.69-0.99) | 0.0401 | 0.85 (0.66-1.09) | 0.2099 |
| Black/Other race vs. white | 1.04 (0.80-1.36) | 0.7722 | 1.02 (0.70-1.49) | 0.9040 |
| Adenocarcinoma vs. SCC | 0.83 (0.71-0.98) | 0.0286 | 0.66 (0.53-0.82) | 0.0002 |
| Regional stage vs. localized | 1.92 (1.59-2.31) | <0.0001 | 2.40 (1.92-2.99) | <0.0001 |
| *Charlson comorbidity score* | | | | |
| 0 | Reference |  | Reference |  |
| 1 | 1.04 (0.89-1.21) | 0.6387 | 1.42 (1.13-1.80) | 0.0031 |
| ≥2 | 1.69 (1.16-2.47) | 0.0066 | 1.47 (0.99-2.18) | 0.0565 |
| Perioperative transfusion | 0.94 (0.75-1.17) | 0.5599 | 1.22 (0.90-1.66) | 0.1946 |
| Radiation | 0.99 (0.85-1.15) | 0.8741 | 0.82 (0.66-1.02) | 0.0691 |
| *SEER registry region* | | | | |
| Northeast | Reference |  | Reference |  |
| Midwest | 0.99 (0.76-1.30) | 0.9544 | 1.54 (0.87-2.73) | 0.1425 |
| South | 0.85 (0.63-1.15) | 0.2919 | 0.98 (0.65-1.47) | 0.9058 |
| West | 0.81 (0.65-1.02) | 0.0680 | 0.89 (0.65-1.22) | 0.4615 |
| *Hospital esophagectomy volume* | | | | |
| Quintile 1: 1-9 | Reference |  | Reference |  |
| Quintile 2: 10-22 | 0.83 (0.66-1.05) | 0.1267 | 0.64 (0.45-0.91) | 0.0123 |
| Quintile 3: 23-49 | 0.62 (0.46-0.84) | 0.0020 | 0.60 (0.39-0.93) | 0.0228 |
| Quintile 4: 50-87 | 0.56 (0.37-0.86) | 0.0077 | 0.46 (0.25-0.84) | 0.0111 |
| Quintile 5: 88-209 | 0.40 (0.26-0.62) | <0.0001 | 0.46 (0.26-0.81) | 0.0076 |
| *Education*^†^ | | | | |
| Q1: 2.5%-13.3% | 1.29 (0.92-1.80) | 0.1411 | 1.53 (0.85-2.78) | 0.1593 |
| Q2: 13.4%-18.4% | 1.09 (0.84-1.42) | 0.5031 | 1.05 (0.68-1.63) | 0.8349 |
| Q3: 18.5%-22.9% | 1.36 (1.06-1.74) | 0.0142 | 1.17 (1.74-1.86) | 0.4995 |
| Q4: 23.0%-45.7% | Reference |  | Reference |  |
| *Income*^‡^ | | | | |
| Q1: $25,717-$46,451 | 0.94 (0.68-1.31) | 0.7283 | 0.84 (0.48-1.46) | 0.5364 |
| Q2: $46,452-$53,263 | 1.01 (0.77-1.33) | 0.9176 | 0.96 (0.61-1.50) | 0.8541 |
| Q3: $53,264-$62,815 | 0.85 (0.61-1.20) | 0.8189 | 1.01 (0.68-1.51) | 0.9519 |
| Q4: $62,816-$91,050 | Reference |  | Reference |  |

TTE, Transthoracic esophagectomy

THE, Transhiatal esophagectomy

SCC, Squamous cell carcinoma

Q, Quartile

^†^Mean % residents in county with college education

^‡^Mean county-level median income
