# Supplementary material for: Surgical approach and the impact of epidural analgesia on survival after esophagectomy for cancer: A population-based retrospective cohort study
Source: PLoS One. 2019 Jan 22;14(1):e0211125. doi: 10.1371/journal.pone.0211125 (PMC6342325; doi:10.1371/journal.pone.0211125)
Supplement: S3 Table — (DOCX) [file pone.0211125.s005.docx]

**S3 Table.** Marginal Cox model for time to death (5-year survival) in adenocarcinoma patients - stratified by propensity score quartiles

| **Variable** | **HR (95%CI)** | **P value** |
| --- | --- | --- |
| *Epidural receipt* | | |
| Among those receiving TTE | 0.80 (0.67-0.96) | 0.0142 |
| Among those receiving THE | 1.00 (0.79-1.27) | 0.9812 |
| *Transhiatal esophagectomy* | | |
| Among those receiving epidural | 0.96 (0.74-1.24) | 0.9604 |
| Among those not receiving epidural | 0.77 (0.59-1.00) | 0.7677 |
| Age at diagnosis | 1.05 (1.03-1.07) | <0.0001 |
| Female gender vs. male | 0.73 (0.59-0.90) | 0.0029 |
| Black/Other race vs. white | 0.89 (0.58-1.36) | 0.5876 |
| Regional stage vs. localized | 2.29 (1.96-2.69) | <0.0001 |
| *Charlson comorbidity score* | | |
| 0 | Reference |  |
| 1 | 1.09 (0.94-1.27) | 0.2674 |
| ≥2 | 1.59 (1.17-2.17) | 0.0034 |
| Perioperative transfusion | 1.03 (0.78-1.36) | 0.8328 |
| Radiation | 0.99 (0.85-1.14) | 0.8427 |
| *SEER registry region* | | |
| Northeast | Reference |  |
| Midwest | 1.03 (0.78-1.36) | 0.8328 |
| South | 0.89 (0.68-1.17) | 0.4111 |
| West | 0.86 (0.69-1.06) | 0.1538 |
| *Hospital esophagectomy volume* | | |
| Quintile 1: 1-9 | Reference |  |
| Quintile 2: 10-22 | 0.82 (0.63-1.06) | 0.1212 |
| Quintile 3: 23-49 | 0.65 (0.48-0.88) | 0.0050 |
| Quintile 4: 50-87 | 0.58 (0.40-0.86) | 0.0065 |
| Quintile 5: 88-209 | 0.47 (0.30-0.76) | 0.0020 |
| *Education*^†^ | | |
| Q1: 2.5%-13.3% | 1.24 (0.90-1.73) | 0.1906 |
| Q2: 13.4%-18.4% | 1.09 (0.83-1.44) | 0.5412 |
| Q3: 18.5%-22.9% | 1.40 (1.08-1.81) | 0.0101 |
| Q4: 23.0%-45.7% | Reference |  |
| *Income*^‡^ | | |
| Q1: $24,869-$46,451 | 1.02 (0.73-1.42) | 0.9225 |
| Q2: $46,452-$52,489 | 1.00 (0.75-1.34) | 0.9955 |
| Q3: $52,490-$62,815 | 1.05 (0.80-1.36) | 0.7423 |
| Q4: $62,816-$91,050 | Reference |  |

TTE, Transthoracic esophagectomy; THE, Transhiatal esophagectomy

SCC, Squamous cell carcinoma

Q, Quartile

^†^Mean % residents in county with college education

^‡^Mean county-level median income
